# Peginterferon plus weight-based ribavirin for treatment-naïve hepatitis C virus genotype 2 patients not achieving rapid virologic response: a randomized trial

**DOI:** 10.1038/srep11710

**Published:** 2015-07-01

**Authors:** Chen-Hua Liu, Chung-Feng Huang, Chun-Jen Liu, Chia-Yen Dai, Jee-Fu Huang, Jou-Wei Lin, Cheng-Chao Liang, Sheng-Shun Yang, Chih-Lin Lin, Tung-Hung Su, Hung-Chih Yang, Pei-Jer Chen, Ding-Shinn Chen, Wan-Long Chuang, Jia-Horng Kao, Ming-Lung Yu

**Affiliations:** 1 Department of Internal Medicine, National Taiwan University Hospital, Taipei, Taiwan; 2 Hepatitis Research Center, National Taiwan University Hospital, Taipei, Taiwan; 3 Graduate Institute of Clinical Medicine, National Taiwan University College of Medicine, Taipei, Taiwan; 4 Department of Internal Medicine, National Taiwan University Hospital, Yun-Lin Branch, Douliou, Taiwan; 5 Institute of Clinical Medicine and Faculty of Internal Medicine, College of Medicine, Kaohsiung Medical University, Kaohsiung, Taiwan; 6 Hepatobiliary Division, Department of Internal Medicine and Hepatitis Center, Kaohsiung Medical University Hospital, Kaohsiung, Taiwan; 7 Department of Occupational Medicine, Kaohsiung Municipal Ta-Tung Hospital, Kaohsiung, Taiwan; 8 Institute of Biomedical Sciences, National Sun Yat-Sen University, Kaohsiung, Taiwan; 9 Department of Internal Medicine, Far Eastern Memorial Hospital, Taipei, Taiwan; 10 Department of Internal Medicine, Taichung Veterans General Hospital, Taichung, Taiwan; 11 Department of Gastroenterology, Taipei City Hospital, Ren-Ai Branch, Taipei, Taiwan; 12 Department of Microbiology, National Taiwan University College of Medicine and National Taiwan University Hospital, Taipei; 13 Genomics Research Center, Academia Sinica, Taiwan

## Abstract

Hepatitis C virus genotype 2 (HCV-2) slow responders poorly respond to 24 weeks of peginterferon (Peg-IFN) plus ribavirin (RBV). We evaluated the efficacy of extended 48-week regimen and the role of interleukin-28B (IL-28B) genotype in this clinical setting. Treatment-naïve HCV-2 patients not achieving rapid virologic response (RVR) by Peg-IFN alfa-2a 180 μg/week plus weight-based RBV (1,000–1,200 mg/day, cutoff body weight of 75 kg) were randomly assigned to receive a total duration of 48 (n = 94) or 24 (n = 93) weeks of therapy. The primary endpoint was sustained virologic response (SVR). Baseline patient characteristics to predict SVR were analyzed. Patients receiving 48 weeks of treatment had a greater SVR rate than those receiving 24 weeks of treatment (70.2% versus 46.2%, *P* = 0.001). Compared to patients treated for 24 weeks, the SVR rate in those treated for 48 weeks increased by 10.9% [95% CI: −5.9% to 27.7%] and 65.6% [95% CI: 44.5% to 86.7%] if they had IL-28B rs8099917 TT genotype, and GT/GG genotype, respectively (interaction *P* = 0.002). In conclusion, 48-week treatment with Peg-IFN plus weight-based RBV provides a greater SVR rate than 24-week treatment in treatment-naïve HCV-2 patients with unfavorable IL-28B genotypes who fail to achieve RVR.

Hepatitis C virus (HCV) infection is the leading cause of cirrhosis, hepatic decompensation, hepatocellular carcinoma (HCC) and liver transplantation^1^. It is estimated that >185 million people with chronic HCV infection worldwide^2^. Although HCV genotype 2 (HCV-2) infection is relatively uncommon in North America and West Europe, it is common in East Asia^2^,^3^,^4^. By peginterferon (Peg-IFN) plus ribavirin (RBV) treatment, patients with HCV-2 infection have higher sustained virologic response (SVR) rates than patients with HCV genotype 1 or 4 (HCV-1/4) infection (75–95% versus 39–79%)^5^,^6^,^7^,^8^,^9^,^10^,^11^,^12^. Recently, the safety and efficacy of sofosbuvir (SOF)-based therapies are excellent for HCV-2 patients, especially for those who are ineligible, intolerant or failure to prior Peg-IFN plus RBV therapy^13^,^14^,^15^,^16^. Although IFN-free regimens provide novel treatment options for these patients, the treatment costs and drug availability preclude the widespread use of these agents^17^.

In patients with HCV-2 infection, those achieving rapid virologic response (RVR) have comparable SVR rates by a truncated (12–16 weeks) or standard (24 weeks) duration of Peg-IFN plus RBV therapy^12^,^18^,^19^. However, in HCV-2 patients not achieving RVR, the benefits of improving the SVR rates by extending the treatment duration from 24 weeks to 36–48 weeks or by using weight-based (1,000–1,200 mg/day) rather than flat (800 mg/day) RBV dosages are controversial^20^,^21^,^22^,^23^. Although the SVR rates in HCV-2 patients achieving RVR by Peg-IFN plus RBV are similar regardless of interleukin-28B (IL-28B) genotypes, the role of IL-28B genotypes in HCV-2 not achieving RVR has not been fully elucidated^23^,^24^,^25^,^26^. Therefore, we aimed to compare the efficacy of Peg-IFN plus weight-based RBV for 24 or 48 weeks in HCV-2 patients not achieving RVR by Peg-IFN plus RBV, and to evaluate the role of IL-28B genotypes on the viral responses in these patients.

## Results

### Patient Characteristics

**Figure 1:**
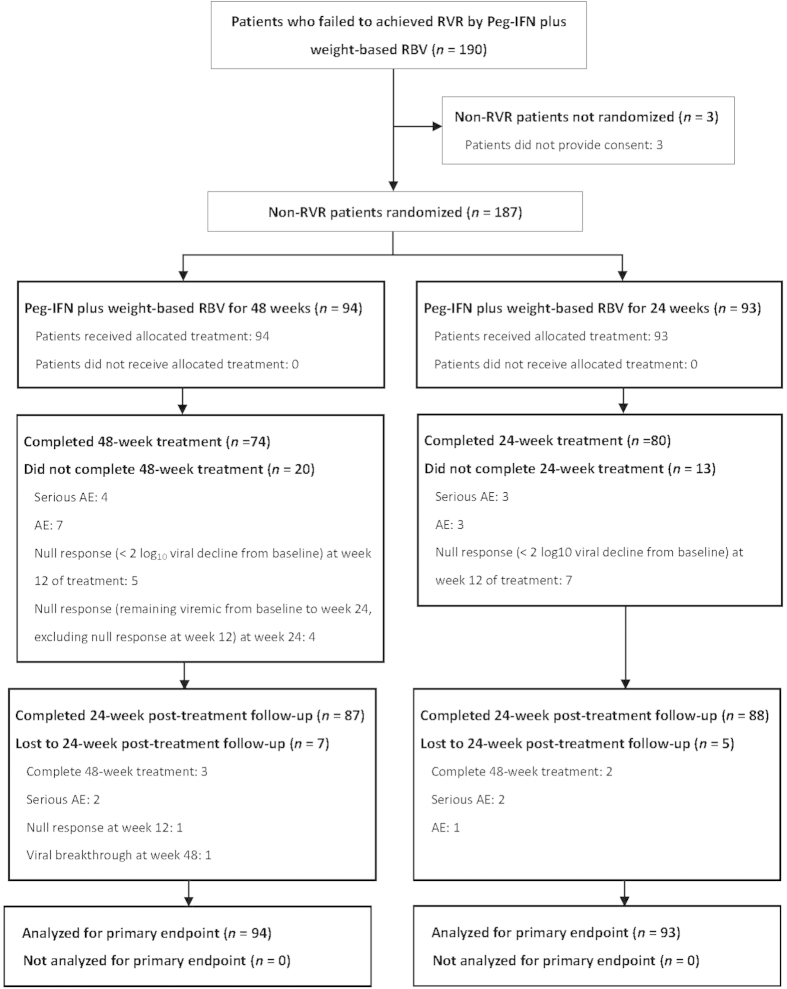
Study Flow Diagram. Peg-IFN: peginterferon, RBV: ribavirin; RVR: rapid virologic response, AE: adverse event.

Among the 190 patients not achieving RVR by Peg-IFN plus weight-based RBV therapy, 187 were allocated to a total duration of 48-week (n = 94) or 24-week (n = 93) therapy. Seventy-four (78.7%) and 80 (86.0%) patients assigned to 48-week and 24-week therapies completed treatment, and 87 (92.6%) and 88 (94.6%) patients completed 24 weeks of post-treatment follow-up to assess the SVR rates (Fig. 1). The baseline patient characteristics were comparable between the two arms (Table 1).They were slim with an average BMI of 26.3–26.7 kg/m^2^.Most of the patients had high baseline viral load (62–65%) and favorable IL-28B rs8099917 genotype (71–74%), and were infected with 2a subtype (68–71%). With regard to aspartate aminotransferase (AST)-to-platelet ratio index (APRI) score, 23.7–27.7% patients had a score of >2.00.

### Efficacy

The early virologic response (EVR) (91.5% versus 88.2%, RD: 3.3% [95% CI: −5.3% to 12.0%]; *P* = 0.45) and the end-of-treatment virologic response (ETVR) (88.3% versus 80.6%, RD: 7.7% [95% CI: −2.7% to 18.0%]; *P* = 0.15) rates were comparable between the treatment arms. The SVR rate of 48-week treatment was greater than 24-week treatment (70.2% versus 46.2%, RD: 24.0% [95% CI: 10.3% to 37.7%]; *P* = 0.001) (Table 2). Patients with RBV cumulative exposure <60% had lower SVR rates than those with RBV cumulative exposure ≥60% in 48-week arm (77.6% versus 38.9%, *P *= 0.003) and 24-week arm (53.8% versus 6.7%, *P *= 0.001), respectively.

### Subgroup Analyses for Prespecified Factors

Differences of SVR rates between patients receiving 48 and 24 weeks of treatment did not vary by baseline viral load (interaction *P* = 0.82), subgenotype (interaction *P* = 0.89), age (interaction *P* = 0.73), sex (interaction *P* = 0.94), body mass index (BMI) (interaction *P* = 0.26), APRI score (interaction *P* = 0.83) or RBV cumulative exposure (interaction *P *= 0.48) (Table 3). Compared to patients with favorable IL-28B rs8099917 genotype (69.2% versus 58.3%, risk difference (RD): 10.9% [95% CI: −5.9% to 27.7%]), patients with unfavorable IL-28B rs8099917 genotypes receiving 48-week therapy achieved a greater SVR rate than 24-week therapy (73.9% versus 8.3%, RD: 65.6% [95% CI: 44.5% to 86.7%]; interaction *P* = 0.002). The SVR rates of 48-week and 24-week treatment were similar when the patients had BMI >30 kg/m^2^ (52.6% versus 50.0%, RD: 2.6% [95% CI: −33.5% to 38.8%]).

### Safety

The constitutional and laboratory adverse events (AEs) were similar between the two arms (Table 4). Four and 3 patients in 48-week and 24-week arms had serious AEs during treatment (4.3% versus 3.2%, RD: 1.1% [95% CI: −4.4% to 6.5%]. The AE-related withdrawal rates were 11.7% in 48-week treatment arm and 6.5% in 24-week treatment arm (RD: 5.2% [95% CI: 2.9% to 13.5%]). The rates of anemia beyond week 6 of treatment were 44.7% and 34.4% (RD: 10.3% [95% CI: −3.7% to 24.2%].

## Discussion

In this study, we demonstrated that 48-week treatment with Peg-IFN plus weight-based RBV provided a greater SVR rate than 24-week treatment (70.2% versus 46.2%) in treatment-naïve HCV-2 patients not achieving RVR. Our results were in line with previous reports from U.S. and Japan showing that the beneficial effects of extending the treatment duration to 36–48 weeks for these patients^21^,^22^. In the N-CORE study, Shiffman *et al*. showed that the SVR rate of 48-week treatment was significantly greater than 24-week treatment if the patients completed a total course of treatment (73% versus 54%, *P* = 0.02). Instead, 48-week treatment did not provide a higher SVR rate than 24-week treatment by intention-to-treat (ITT) analysis (61% versus 52%, *P* = 0.19)^21^. Compared to patients receiving 24 weeks of treatment, the SVR rate of 48-week treatment in our study was greater than that in the N-CORE study (46.2% versus 52% for 24 weeks; 70.2% versus 61% for 48 weeks). Although patients with RBV cumulative exposure <60% had lower SVR rates than those with RBV cumulative exposure ≥60%, 48-week treatment provided greater SVR rates than 24-week treatment regardless of the grades of RBV cumulative exposure. Our findings were in line with the N-CORE study that RBV dosage and treatment duration may improve the SVR rates in HCV-2 patients not achieving RVR by reducing the relapse rates, especially for those receiving weight-based RBV for 48 weeks^21^.

We further analyzed if any pre-specified patient characteristics could help the health-care providers determine the optimized treatment strategies in HCV-2 patients not achieving RVR. In patients with favorable IL-28B genotype, the SVR rate of 48-week treatment was not superior to 24-week treatment. In contrast, extending the treatment duration to 48 weeks provided a superior SVR rate to standard 24 weeks of treatment if patients had unfavorable IL-28B genotypes. Similar results were also observed among HCV-1 slow responders that only those carrying unfavorable IL-28B genotypes could benefit from extended therapy to 72 week^27^. However, Yamaguchi *et al*. showed that regardless of treatment duration, the IL-28B genotypes did not affect the SVR rates in these patients. The discrepancy of the results may be reasoned by the design of the studies. Whereas we randomized 187 patients as an 1:1 ratio to maximize the power of the study, Yamaguchi *et al*. allowed 59 patients 24 (n = 40) or 48 (n = 19) weeks of treatment on patients’ preference, which might introduce selection bias and result in insufficient power to discriminate the effects of extended duration of therapy^23^.

In Asian HCV-2 patients where the prevalence rate of favorable IL-28B genotype is high, 24-week treatment would be sufficient to provide an overall SVR rate of 58.3% for these patients^26^. In HCV-2 patients of non-Asian ancestry where the prevalence rate of favorable IL-28B genotype is relatively low, the patients may extend the treatment duration to 48 weeks to secure the high SVR rate (73.9%) or may switch to sofosbuvir-based therapies if they were intolerable or unwilling to receiving continuous Peg-IFN plus RBV therapy in countries where sofosbuvir is available^13^,^14^,^15^,^16^.

The EASL clinical practice guideline and the expert’s opinions recommended that HCV-2 patients with cirrhosis and with BMI >30 kg/m^2^ should receive 24–48 weeks of Peg-IFN plus RBV regardless of RVR^17^,^28^. In contrast to the improved SVR rates of 48-week treatment irrespective of the severity of hepatic fibrosis, extending the treatment duration to 48 weeks would be beneficial to our patients with BMI ≤30 kg/m^2^. On the basis of our findings, HCV-2 patients with IL-28B favorable genotype or with BMI >30 kg/m^2^ should not receive extended duration of dual therapy if they failed to achieve RVR.

With regard to the safety, our data showed that although the AE-related withdrawal rates were greater in 48-week treatment arm than in 24-week treatment arm, the constitutional and laboratory AE rates were comparable between two arms, and the serious AE rates were low in both arms. The health care providers can safely treat these patients for 24 or 48 weeks based on IL-28B genotypes, but care must be taken to keep the RBV cumulative exposure ≥60% to secure the SVR rates^5^,^6^,^7^,^8^,^9^,^20^,^21^,^22^,^23^.

Although we demonstrated that the SVR rate of 48-weeks treatment with Peg-IFN plus weight-based RBV was greater than that of 24-weeks of treatment. Several limitations existed. First, the effect of IL-28B genotypes on SVR was not prospectively randomized to analysis, which might cause observer bias. However, the distributions of IL-28B genotypes between the two treatment arms were similar, which minimized the potential errors of our findings. Second, the diagnosis for fibrosis and cirrhosis was based on APRI, and not on liver histology.

In conclusion, 48-week treatment with Peg-IFN plus weight-based RBV provides a greater SVR rate than 24-week treatment for treatment-naïve HCV-2 patients not achieving RVR, especially for those with unfavorable IL-28B genotypes. HCV-2 patients with favorable IL-28B genotype may receive 24 weeks of treatment without compromising the SVR rates.

## Methods

### Patients

From 2007 to 2013, treatment-naïve patients with HCV-2 infection who were aged ≥18 years and had serum alanine aminotransferase (ALT) level ≥upper limit of normal (ULN) who failed to achieve RVR by Peg-IFN plus RBV were consecutively enrolled in the Tailored Regimens of Peginterferon alfa-2a and Ribavirin for Genotype 2 Chronic Hepatitis C Patients (TARGET-2) trial in 8 academic centers in Taiwan. Chronic HCV infection was defined as presence of anti-HCV antibody (Abbott HCV EIA 3.0, Abbott Laboratories, Abbott Park, Illinois, USA) and HCV RNA (Cobas TaqMan HCV Test v2.0, Roche Diagnostics GmbH, Mannheim, Germany, limit of detection: 15 IU/mL) for ≥6 months. HCV-2 infection was confirmed by reverse hybridization assay (Versant HCV Genotype 2.0 assay, Siemens Healthcare Diagnostics, Illinois, USA)^29^.

Before receiving treatment, patients were excluded if they had anemia (hemoglobin levels <13 g/dL for men or <12 g/dL for women), neutropenia (neutrophil count <1.5 × 10^9^ cells/L), thrombocytopenia (platelet count <90 × 10^9^ cells/L), non HCV-2 infection, hepatitis B virus (HBV) or human immunodeficiency virus (HIV) co-infection, daily alcohol consumption >20 g, serum albumin level <35 g/L, serum bilirubin level ≥1.5 times ULN, serum AST or ALT level ≥10 times ULN, serum creatinine level ≥1.5 times ULN, decompensated cirrhosis, autoimmune liver diseases, neoplastic diseases, immunosuppressive therapy, drug abuse, pregnancy, poorly controlled autoimmune diseases, cardiopulmonary diseases, neuropsychiatric diseases, and diabetes mellitus with retinopathy or were unwilling to receive contraception during the study period.

The protocol was approved by Taiwan Joint Institutional Review Board and was conducted in accordance with the principles of Declaration of Helsinki and the International Conference on Harmonization for Good Clinical Practice. All patients provided written informed consent before participation in the study.

### Study Design

**Figure 2:**
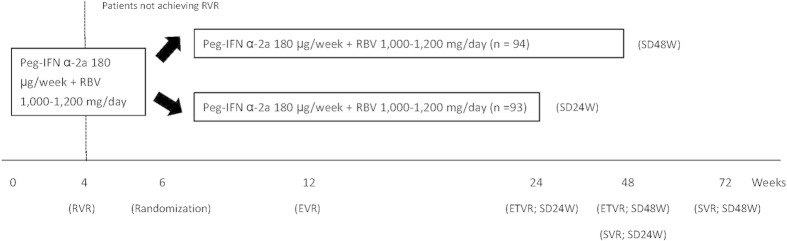
Study Design. Peg-IFN: peginterferon, RBV: ribavirin; RVR: rapid virologic response, ETV: early virologic response, ETVR: end-of-treatment virologic response, SVR: sustained virologic response, SD: weight-based dosing, W: week.

This was a multicenter, open-label, randomized trial. All eligible patients received combination therapy of Peg-IFN alfa-2a 180 μg/week (Pegasys, Hoffman-LaRoche, Basel, Switzerland) plus weight-based RBV 1,000–1,200 mg/day (Copegus, Hoffman-LaRoche, Basel, Switzerland; 1,000 mg/day for patients weighted <75 kg and 1,200 mg/day for patients weighted ≥75 kg). Patients who failed to achieve RVR were randomly assigned as 1:1 ratio at week 6 of treatment to receive total treatment duration of 48 or 24 weeks (Fig. 2). Randomization was computer-generated in blocks of 4 with centralized allocation and the code was secured by an independent assistant.

Baseline demographic data, hemogram, biochemical assays (serum albumin, bilirubin, AST, ALT, and creatinine), serologic assays (anti-HCV, HBsAg, and anti-HIV), virologic assays (HCV RNA and HCV genotype) were evaluated before enrollment. Because our study was conducted before the discovery of IL-28B genetic polymorphisms, we added the human genomic assay for IL-28B rs8099917 genotypes (ABI TaqMan allelic discrimination kit and ABI7900HT Sequence Detection System, Applied Biosystems, Life Technologies Corporation, Grand Island, New York, USA) after 2009 to evaluate the effects of these genetic polymorphisms on responses to therapy. The stage of hepatic fibrosis was assessed by APRI^30^. The baseline viral load was defined as high or low level at a cutoff value of 800,000 IU/mL^11^. Favorable IL-28B rs8099917 genotype was defined as patients with homozygous TT genotype, whereas unfavorable genotype was defined as patients with heterozygous GT or homozygous GG genotype^31^,^32^,^33^. Significant hepatic fibrosis (≥F2 by Metavir score) and cirrhosis (F4 by Metavir score) were defined an APRI score of >1.50 and >2.00, respectively^34^.

### Efficacy

All patients received treatment for 48 or 24 weeks and posttreatment follow-up for 24 weeks. Serial serum HCV RNA levels were assessed at weeks 12, 24, 48 (for patients assigned to 48 weeks of treatment) of treatment, and at week 24 posttreatment. The on-treatment virologic responses (including EVR, and ETVR), and the posttreatment virologic response (including SVR), were defined as previously described^11^. Patients who failed to achieve EVR (null response at week 12), who achieved EVR but remained viremic at week 24 of treatment (null response at week 24) or who had viral breakthrough during treatment were considered nonresponders and treatment was stopped. For patients who prematurely discontinued treatment, the ETVR was assessed at the time of treatment discontinuation.

The primary endpoint was SVR, defined as undetectable serum HCV RNA 24 weeks off-therapy. Patients who were null-responsive to treatment or who had viral breakthrough during treatment were considered not to have achieved SVR, regardless of the HCV RNA data at the end of follow-up. Patients who relapsed after the treatment and who lacked the end of follow-up data to assess SVR were also considered not to have achieved SVR.

### Safety

The investigators used a prespecified checklist to assess the severity and the causality of the constitutional and laboratory AEs at weeks 6 and 8 of treatment and then every 4 weeks until the end of follow-up. The severity of all AEs was graded according to the Common Terminology Criteria for Adverse Events (CTCAE), version 3.0. The safety summary was assessed for all patients from randomization to the last visit. Patients were withdrawn from the study if they developed serious AEs, missed receiving the allocated treatment >4 consecutive weeks, or subjectively requested to stop treatment.

The Peg-IFN alfa-2a was reduced in a stepwise dosage of 45 μg/week according to the severity of constitutional AEs. Dosages were also reduced from 180 μg/week to 90 μg/week or treatment was stopped according to laboratory AEs (the dosage was reduced to 90 μg/week if the neutrophil count was <0.75 × 10^9^ cells/L or the platelet count was <50 × 10^9^ cells/L; treatment was stopped if the neutrophil count was <0.50 × 10^9^ cells/L or the platelet count was <25 × 10^9^ cells/L). RBV was reduced in a stepwise dosage of 200 mg/day if the hemoglobin level was <10 g/dL, and was stopped if the hemoglobin level was <8.5 g/dL. The erythropoiesis-stimulating agent (ESA) was not permitted in the study. Blood transfusion was permitted for patients who developed serious AEs. If the constitutional or laboratory AEs improved or resolved after dose reduction or treatment cessation, a return to the lowest dosage of the study medications was permitted.

### Statistical Analyses

Data were analyzed by Statistical Program for Social Sciences (SPSS 17.0; SPSS Inc., Chicago, Illinois, USA). On the basis of the assumption that the SVR rate was 53% for patients who received 24 weeks of treatment, we estimated that a total of 182 patients would provide 90% power to detect an absolute increase in SVR for patients who received 48 weeks of treatment of 21% points or more (2-sided α = 0.05). Patient characteristics were expressed as mean (standard deviation, SD) and percentage when appropriate.

The proportions for viral response between treatment arms were compared and reported by RD. *P* values for RD were obtained by the Wald asymptotic test. The subgroup analyses based on the prespecified factors to predict SVR, including baseline viral load, subgenotype, IL-28B rs8099917 genotype, age, sex, BMI, APRI score, and RBV cumulative exposure, were compared by RD. The interactions between the prespecified factors and the allocated treatment were tested by the stratified Mantel-Haenszel test. All statistical tests were two-tailed and the results were statistically significant when a *P* value was <0.05.

## Additional Information

**How to cite this article**: Liu, C.-H. *et al*. Peginterferon plus weight-based ribavirin for treatment-naïve hepatitis C virus genotype 2 patients not achieving rapid virologic response: a randomized trial. *Sci. Rep*. **5**, 11710; doi: 10.1038/srep11710 (2015).

## Figures and Tables

**Table 1:** Baseline Patient Characteristics*.

Characteristics	SD48W, N = 94	SD24W, N = 93
Mean age (SD), y	56 (12)	56 (13)
Age >50 y	64 (68)	59 (63)
Male sex	51 (54)	52 (56)
Mean weight (SD), kg	69 (13)	69 (11)
Mean BMI (SD), kg/m^2^	26.7 (4.5)	26.3 (3.5)
BMI (SD), kg/m^2^		
≤27.0	54 (57.4)	52 (55.9)
27.1–30.0	21 (22.3)	29 (31.2)
>30.0	19 (20.2)	12 (12.9)
Mean hemoglobin level (SD), g/dL	14.4 (1.5)	14.7 (1.4)
Mean white cell count (SD), 10^9^cells/L	5.6 (1.4)	5.8 (1.5)
Mean neutrophil count (SD), 10^9^cells/L	3.0 (1.1)	3.2 (1.2)
Mean platelet count (SD), 10^9^cells/L	175 (61)	182 (64)
Mean albumin level (SD), g/dL	4.3 (0.4)	4.3 (0.3)
Mean total bilirubin level (SD), mg/dL	1.0 (0.4)	1.0 (0.5)
Mean AST quotient (SD), ULN	2.2 (1.2)	2.3 (1.1)
Mean ALT quotient (SD), ULN	3.4 (2.1)	3.8 (2.3)
Mean APRI score (SD)	1.5 (1.0)	1.5 (1.1)
APRI score		
≤1.50	56 (59.6)	50 (53.8)
1.51–2.0	12 (12.7)	21 (22.5)
>2.00	26 (27.7)	22 (23.7)
Mean HCV RNA level (SD), log_10_ IU/mL	6.0 (0.9)	6.0 (0.6)
HCV RNA level, IU/mL		
≤800,000	33 (35)	35 (38)
>800,000	61 (65)	58 (62)
Subgenotype		
2a	64 (68)	66 (71)
2b	24 (26)	20 (22)
2a + 2b	6 (6)	7 (8)
IL-28B rs8099917 genotype†		
TT	65 (74)	60 (71)
GT and GG	23 (26)	24 (29)

SD: standard deviation, BMI: body mass index, AST: aspartate aminotransferase, ALT: alanine aminotransferase, ULN: upper limit of normal, APRI: aspartate aminotransferase to platelet ratio index, HCV: hepatitis C virus, IU: international unit, IL: interleukin, SD48W: 48 weeks of peginterferon alfa-2a plus weight-based ribavirin, SD24W: 24 weeks of peginterferon alfa-2a plus weight-based ribavirin.

^*^Values are numbers (percentages) unless otherwise indicated.

^†^Available number of patients (%) for analysis: 88 (94%) and 84 (90%) in SD48W and SD24W arms, respectively.

**Table 2:** On-treatment and Off-treatment Virologic Responses.

Variable	SD48W, n/N (%)	SD24W, n/N (%)	RD (95% CI)	*P* value*
On-treatment virologic response				
EVR	86/94 (91.5)	82/93 (88.2)	3.3 (−5.3 to 12.0)	0.45
ETVR	83/94 (88.3)	75/93 (80.6)	7.7 (−2.7 to 18.0)	0.15
Virologic outcome				
SVR†	66/94 (70.2)	43/93 (46.2)	24.0 (10.3 to 37.7)	0.001
Non-SVR	28/94 (29.8)	50/93 (53.8)		
Relapse	12/94 (12.8)	28/93 (30.1)		
Null-response‡	9/94 (9.6)	12/93 (12.9)		
Viral breakthrough§	2/94 (2.1)	5/93 (5.4)		
Undetermined||	5/94 (5.3)	5/93 (5.4)		

RD; risk reduction, EVR: early virologic response, ETVR: end-of-treatment virologic response, SVR: sustained virologic response, CI: confidence interval.

^*^P values were obtained by Wald asymptotic test.

^†^Patients who were lost to 24-week follow-up, were null-responsive to treatment, or had viral breakthrough or relapsed after treatment were considered failure to achieve SVR.

^‡^SD48W arm: 5 patients failed to achieve EVR; 4 patients achieved EVR but remained viremic at week 24 of treatment. SD24W arm: 7 patients failed to achieve ETR; 5 patients achieved EVR but remained viremic at week 24 of treatment.

^§^SD48W arm: 2 patients had viral breakthrough at week 48 of treatment. SD24W arm: 5 patients had viral breakthrough at week 24 of treatment.

^||^All patients lost to 24-week post-treatment follow-up, and all had undetectable HCV RNA level at the time of treatment discontinuation. SD48W arm: 3 completed 48 weeks of treatment, and 2 prematurely discontinued treatment at weeks 36 and 40 due to serious adverse events. SD24W arm: 2 completed 24 weeks of treatment, 2 prematurely discontinued treatment at weeks 12 and 16 due to serious adverse events and 1 prematurely discontinued treatment at week 12 due to constitutional adverse event.

**Table 3:** Subgroup Analyses of Prespecified Factors for SVR.

Variable	SD48W, n/N (%)	SD24W, n/N (%)	RD (95% CI)	*P*value for interaction*
Baseline viral load				0.82
≤800,000 IU/mL	27/33 (81.8)	21/35 (60.0)	21.8 (0.9 to 42.7)	
>800,000 IU/mL	39/61 (63.9)	22/58 (37.9)	26.0 (8.7 to 43.3)	
Subgenotype				0.89
2a	44/64 (68.8)	31/66 (45.5)	21.8 (5.2 to 38.3)	
2b	18/24 (75.0)	10/20 (50.0)	25.0 (−2.9 to 52.9)	
2a+2b	4/6 (67.7)	3/7 (42.9)	23.8 (−28.8 to 76.4)	
IL-28B rs8099917 genotype†				0.002
TT	45/65 (69.2)	35/60 (58.3)	10.9 (−5.9 to 27.7)	
GT and GG	17/23 (73.9)	2/24 (8.3)	65.6 (44.5 to 86.7)	
Age				0.73
≤50 y	23/30 (76.7)	17/34 (50.0)	26.7 (4.1 to 49.3)	
>50 y	43/64 (67.2)	26/59 (44.1)	23.1 (6.1 to 40.2)	
Sex				0.94
Female	30/43 (69.8)	19/41 (46.3)	23.4 (2.9 to 44.0)	
Male	36/51 (70.6)	24/52 (46.2)	24.4 (6.0 to 43.0)	
BMI				0.26
≤27.0 kg/m^2^	41/54 (75.9)	25/52 (48.1)	27.9 (10.1 to 45.6)	
27.1–30.0 kg/m^2^	15/21 (71.4)	12/29 (41.4)	30.1 (3.7 to 56.4)	
>30.0 kg/m^2^	10/19 (52.6)	6/12 (50.0)	2.6 (−33.5 to 38.8)	
APRI score				0.83
≤1.50	38/56 (67.9)	23/50 (46.0)	21.9 (3.4 to 40.3)	
1.51–2.00	9/12 (75.0)	9/21 (42.9)	32.1 (−0.2 to 64.5)	
>2.00	19/26 (73.1)	11/22 (50.0)	23.1 (−3.9 to 50.0)	
RBV cumulative exposure				0.48
<60%	7/18 (38.9)	1/15 (6.7)	32.2 (6.4 to 58.4)	
60%–80%	15/19 (77.8)	9/20 (45.0)	34.0 (5.5 to 62.4)	
≥80%	44/57 (77.2)	33/58 (56.9)	20.3 (3.5 to 37.1)	

RD: risk reduction, CI: confidence interval, IL: interleukin, BMI: body mass index, APRI: aspartate aminotransferase to platelet ratio index

^*^The interaction for the prespecified factors was compared by stratified Mantel-Haenszel test.

^†^Available number of patients (%) for analysis: 88 (94%) and 84 (90%) in SD48W and SD24W arms, respectively.

**Table 4:** Adverse Events, Dose reduction and Treatment Discontinuation in Treated Patients *.

Parameter	SD48W, N = 94	SD24W, N = 93
Serious AEs		
All†	4 (4.3)	3 (3.2)
Death	0 (0)	0 (0)
Treatment-related	3 (3.2)	3 (3.2)
Treatment withdrawal due to AEs	11 (11.7)	6 (6.5)
Dose reduction to AEs		
Peginterferon	23 (24.5)	20 (21.5)
Ribavirin	45 (47.9)	35 (37.6)
Constitutional AEs		
Flu-like symptoms	29 (30.9)	27 (29.0)
Fatigue	57 (60.6)	54 (58.1)
Headache	26 (27.7)	28 (30.1)
Insomnia	34 (36.2)	33 (35.5)
Irritability	11 (11.7)	10 (10.8)
Depression	14 (14.9)	12 (12.9)
Anorexia	30 (31.9)	27 (29.0)
Diarrhea	12 (12.8)	12 (12.9)
Constipation	11 (11.7)	8 (8.6)
Cough	14 (14.9)	15 (16.1)
Dermatitis	28 (29.8)	25 (26.9)
Injection site reaction	15 (16.0)	13 (14.0)
Hair loss/alopecia	25 (26.6)	24 (25.8)
Laboratory AEs‡		
Anemia (week 1–6)§	7 (7.4)	6 (6.5)
Hemoglobin level: 8.5–9.9 g/dL	6 (6.4)	5 (5.4)
Hemoglobin level: <8.5 g/dL	1 (1.1)	1 (1.1)
Anemia (week 6 to end-of-treatment)§	42 (44.7)	32 (34.4)
Hemoglobin level: 8.5–9.9 g/dL	27 (28.7)	22 (23.7)
Hemoglobin level: <8.5 g/dL	15 (16.0)	10 (10.8)
Neutropenia	17 (18.1)	14 (15.1)
Neutrophil count: 0.500–0.749 × 10^9^ cells/L	13 (13.8)	11 (11.8)
Neutrophil count: <0.500 × 10^9^ cells/L	4 (4.3)	3 (3.2)
Thrombocytopenia	9 (9.6)	11 (11.8)
Platelet count: 25–49 × 10^9^ cells/L	8 (8.5)	10 (10.8)
Platelet count: <25 × 10^9^ cells/L	1 (1.1)	1 (1.1)
ALT elevation		
>2 times ULN	16 (17.0)	18 (19.4)
>5 times ULN	2 (2.1)	3 (3.2)
Total bilirubin elevation¶		
>2 mg/dL	5 (5.3)	4 (4.3)
>5 mg/dL	0 (0.0)	0 (0.0)

AE: adverse event, ALT: alanine aminotransferase, ULN: upper limit of normal.

^*^Values are numbers (percentages).

^†^SD48W arm: spontaneous bacterial peritonitis at week 36 of treatment, cellulitis at week 20 of treatment, severe anemia with postural dizziness at week 12 of treatment (hemoglobin level: 7.1 g/dL), and multiple myeloma at week 40 of treatment. SD24W arm: urinary tract infection at week 12 of treatment, community-acquired pneumonia at week 16 of treatment, severe anemia with postural dizziness at week 16 of treatment (hemoglobin level: 7.3 g/dL), respectively. All except the patients developing multiple myeloma were considered treatment-related.

^‡^The grading of the laboratory AEs was shown for patients with the on-treatment nadir level.

^§^Anemia was defined as a nadir hemoglobin level <10.0 g/dL.

^¶^No patient with total bilirubin elevation had concomitant ALT elevation >5 times ULN.

## References

[b1] Rosen H. R. Clinical practice. Chronic hepatitis C infection. N. Engl. J. Med. 364, 2429–2438 (2011). 21696309 10.1056/NEJMcp1006613

[b2] Messina J. P. . Global distribution and prevalence of hepatitis C virus genotypes. Hepatology. 61, 77–87 (2015). 25069599 10.1002/hep.27259 PMC4303918

[b3] Yu M. L. & Chuang W. L. Treatment of chronic hepatitis C in Asia: when East meets West. J. Gastroenterol. Hepatol. 24, 336–345 (2009). 19335784 10.1111/j.1440-1746.2009.05789.x

[b4] Liu C. H. & Kao J. H. Nanomedicines in the treatment of hepatitis C virus infection in Asian patients: optimizing use of peginterferon alfa. Int. J. Nanomedicine. 9, 2051–2067 (2014). 24812506 10.2147/IJN.S41822 PMC4008289

[b5] Manns M. P. . Peginterferon alfa-2b plus ribavirin compared with interferon alfa-2b plus ribavirin for initial treatment of chronic hepatitis C: a randomised trial. Lancet. 358, 958–965 (2001). 11583749 10.1016/s0140-6736(01)06102-5

[b6] Fried M. W. . Peginterferon alfa-2a plus ribavirin for chronic hepatitis C virus infection. N. Engl. J. Med. 347, 975–982 (2002). 12324553 10.1056/NEJMoa020047

[b7] Hadziyannis S. J. . Peginterferon-alpha2a and ribavirin combination therapy in chronic hepatitis C: a randomized study of treatment duration and ribavirin dose. Ann. Intern. Med. 140, 346–355 (2004). 14996676 10.7326/0003-4819-140-5-200403020-00010

[b8] McHutchison J. G. . Peginterferon alfa-2b or alfa-2a with ribavirin for treatment of hepatitis C infection. N. Engl. J. Med. 361, 580–593 (2009). 19625712 10.1056/NEJMoa0808010

[b9] Shiffman M. L. . Peginterferon alfa-2a and ribavirin for 16 or 24 weeks in HCV genotype 2 or 3. N. Engl. J. Med. 357, 124–134 (2007). 17625124 10.1056/NEJMoa066403

[b10] Yu M. L. . Rapid virological response and treatment duration for chronic hepatitis C genotype 1 patients: a randomized trial. Hepatology. 47, 1884–1893 (2008). 18508296 10.1002/hep.22319

[b11] Liu C. H. ., Pegylated interferon-alpha-2a plus ribavirin for treatment-naive Asian patients with hepatitis C virus genotype 1 infection: a multicenter, randomized controlled trial. Clin. Infect. Dis. 47, 1260–1269 (2008). 18834319 10.1086/592579

[b12] Yu M. L. . A randomised study of peginterferon and ribavirin for 16 versus 24 weeks in patients with genotype 2 chronic hepatitis C. Gut. 56, 553–559 (2007). 16956917 10.1136/gut.2006.102558 PMC1856839

[b13] Jacobson I. M. . Sofosbuvir for hepatitis C genotype 2 or 3 in patients without treatment options. N. Engl. J. Med. 368, 1867–1877 (2013). 23607593 10.1056/NEJMoa1214854

[b14] Lawitz E. . Sofosbuvir for previously untreated chronic hepatitis C infection. N. Engl. J. Med. 368, 1878–1887 (2013). 23607594 10.1056/NEJMoa1214853

[b15] Zeuzem S. . Sofosbuvir and ribavirin in HCV genotypes 2 and 3. N. Engl. J. Med. 370, 1993–2001 (2014). 24795201 10.1056/NEJMoa1316145

[b16] Sulkowski M. S. . Daclatasvir plus sofosbuvir for previously treated or untreated chronic HCV infection. N. Engl. J. Med. 370, 211–221 (2014). 24428467 10.1056/NEJMoa1306218

[b17] European Association for Study of Liver. EASL Clinical Practice Guidelines: management of hepatitis C virus infection. J. Hepatol. 60, 392-420 (2014). 24331294 10.1016/j.jhep.2013.11.003

[b18] Di Martino V. . Response-guided peg-interferon plus ribavirin treatment duration in chronic hepatitis C: meta-analyses of randomized, controlled trials and implications for the future. Hepatology. 54, 789–800 (2011). 21674553 10.1002/hep.24480

[b19] Chou R. . Comparative effectiveness of antiviral treatment for hepatitis C virus infection in adults: a systematic review. Ann. Intern. Med. 158, 114–123 (2013). 23437439 10.7326/0003-4819-158-2-201301150-00576

[b20] Sato K. . Response-guided peginterferon-alpha-2b plus ribavirin therapy for chronic hepatitis C patients with genotype 2 and high viral loads. Hepatol. Res. 42, 854–863 (2012). 22487210 10.1111/j.1872-034X.2012.00997.x

[b21] Shiffman M. L. . Extended treatment with pegylated interferon alfa/ribavirin in patients with genotype 2/3 chronic hepatitis C who do not achieve a rapid virological response: final analysis of the randomised N-CORE trial. Hepatol. Int. 8, 517–526 (2014). 10.1007/s12072-014-9555-3 26202757

[b22] Abe H. . New proposal for response-guided peg-interferon-plus-ribavirin combination therapy for chronic hepatitis C virus genotype 2 infection. J. Med. Virol. 85, 1523–1533 (2013). 23775277 10.1002/jmv.23626

[b23] Yamaguchi Y. . Response-guided therapy for patients with chronic hepatitis who have high viral loads of hepatitis C virus genotype 2. Hepatol. Res. 42, 549–557 (2012). 22321126 10.1111/j.1872-034X.2011.00956.x

[b24] Mangia A. . An IL28B polymorphism determines treatment response of hepatitis C virus genotype 2 or 3 patients who do not achieve a rapid virologic response. Gastroenterology. 139, 821–827, 827.e1 (2010). 20621700 10.1053/j.gastro.2010.05.079

[b25] Sarrazin C. . Importance of IL28B gene polymorphisms in hepatitis C virus genotype 2 and 3 infected patients. J. Hepatol. 54, 415–421 (2011). 21112657 10.1016/j.jhep.2010.07.041

[b26] Yu M. L. . Role of interleukin-28B polymorphisms in the treatment of hepatitis C virus genotype 2 infection in Asian patients. Hepatology. 53, 7–13 (2011). 21254157 10.1002/hep.23976

[b27] Scherzer T. M. . Impact of IL28B on treatment outcome in hepatitis C virus G1/4 patients receiving response-guided therapy with peginterferon alpha-2a (40KD)/ribavirin. Hepatology. 54, 1518–1526 (2011). 22006276 10.1002/hep.24546

[b28] Mangia A. & Andriulli A. Tailoring the length of antiviral treatment for hepatitis C. Gut. 59, 1–5 (2010). 20007952 10.1136/gut.2009.179606

[b29] Liu C. H. . Comparison of Abbott RealTime HCV genotype II with VERSANT line probe assay 2.0 for hepatitis C virus genotyping. J. Clin. Microbiol. 53, 1754–1757 (2015). 25740780 10.1128/JCM.03548-14 PMC4400777

[b30] Liu C. H. . Noninvasive tests for the prediction of significant hepatic fibrosis in hepatitis C virus carriers with persistently normal alanine aminotransferases. Liver. Int. 26, 1087–1094 (2006). 17032409 10.1111/j.1478-3231.2006.01355.x

[b31] Huang C. F. . Interleukin-28B genetic variants in identification of hepatitis C virus genotype 1 patients responding to 24 weeks peginterferon/ribavirin. J. Hepatol. 56, 34–40 (2012). 21703176 10.1016/j.jhep.2011.03.029

[b32] Liu C. H. . Interleukin 28B genetic polymorphisms and viral factors help identify HCV genotype-1 patients who benefit from 24-week pegylated interferon plus ribavirin therapy. Antivir. Ther. 17, 477–484 (2012). 22301466 10.3851/IMP2026

[b33] Liu C. H. . Interleukin 28B genetic polymorphisms play a minor role in identifying optimal treatment duration in HCV genotype 1 slow responders to pegylated interferon plus ribavirin. Antivir. Ther. 17, 1059–1067 (2012). 22898703 10.3851/IMP2322

[b34] Wai C. T. . A simple noninvasive index can predict both significant fibrosis and cirrhosis in patients with chronic hepatitis C. Hepatology. 38, 518–526 (2003). 12883497 10.1053/jhep.2003.50346

